# Mutation in *LIM2* Is Responsible for Autosomal Recessive Congenital Cataracts

**DOI:** 10.1371/journal.pone.0162620

**Published:** 2016-11-04

**Authors:** Bushra Irum, Shahid Y. Khan, Muhammad Ali, Haiba Kaul, Firoz Kabir, Bushra Rauf, Fareeha Fatima, Raheela Nadeem, Arif O. Khan, Saif Al Obaisi, Muhammad Asif Naeem, Idrees A. Nasir, Shaheen N. Khan, Tayyab Husnain, Sheikh Riazuddin, Javed Akram, Allen O. Eghrari, S. Amer Riazuddin

**Affiliations:** 1 The Wilmer Eye Institute, Johns Hopkins University School of Medicine, Baltimore, MD, 21287, United States of America; 2 National Centre of Excellence in Molecular Biology, University of the Punjab, Lahore, 53700, Pakistan; 3 King Khaled Eye Specialist Hospital, Riyadh, 12329, Saudi Arabia; 4 Allama Iqbal Medical College, University of Health Sciences, Lahore, 54550, Pakistan; 5 National Centre for Genetic Diseases, Shaheed Zulfiqar Ali Bhutto Medical University, Islamabad, 44000, Pakistan; 6 McKusick-Nathans Institute of Genetic Medicine, Johns Hopkins University School of Medicine, Baltimore, MD, 21205, United States of America; Tsinghua University School of Life Sciences, CHINA

## Abstract

**Purpose:**

To identify the molecular basis of non-syndromic autosomal recessive congenital cataracts (arCC) in a consanguineous family.

**Methods:**

All family members participating in the study received a comprehensive ophthalmic examination to determine their ocular phenotype and contributed a blood sample, from which genomic DNA was extracted. Available medical records and interviews with the family were used to compile the medical history of the family. The symptomatic history of the individuals exhibiting cataracts was confirmed by slit-lamp biomicroscopy. A genome-wide linkage analysis was performed to localize the disease interval. The candidate gene, *LIM2* (lens intrinsic membrane protein 2), was sequenced bi-directionally to identify the disease-causing mutation. The physical changes caused by the mutation were analyzed *in silico* through homology modeling, mutation and bioinformatic algorithms, and evolutionary conservation databases. The physiological importance of LIM2 to ocular development was assessed *in vivo* by real-time expression analysis of *Lim2* in a mouse model.

**Results:**

Ophthalmic examination confirmed the diagnosis of nuclear cataracts in the affected members of the family; the inheritance pattern and cataract development in early infancy indicated arCC. Genome-wide linkage analysis localized the critical interval to chromosome 19q with a two-point logarithm of odds (LOD) score of 3.25. Bidirectional sequencing identified a novel missense mutation, c.233G>A (p.G78D) in *LIM2*. This mutation segregated with the disease phenotype and was absent in 192 ethnically matched control chromosomes. *In silico* analysis predicted lower hydropathicity and hydrophobicity but higher polarity of the mutant *LIM2*-encoded protein (MP19) compared to the wild-type. Moreover, these analyses predicted that the mutation would disrupt the secondary structure of a transmembrane domain of MP19. The expression of *Lim2*, which was detected in the mouse lens as early as embryonic day 15 (E15) increased after birth to a level that was sustained through the postnatal time points.

**Conclusion:**

A novel missense mutation in *LIM2* is responsible for autosomal recessive congenital cataracts.

## Introduction

Cataracts are opacifications that occur in the ocular lens, and are the leading cause of vision loss in children globally [1,2]. Cataracts can be morphologically diverse and classification systems are frequently based on the location of opacity. Congenital cataracts can be divided by these traits: total (mature or complete), polar (anterior or posterior), zonular (nuclear, lamellar, or sutural), and capsular or membranous [3].

Congenital cataracts are also genetically heterogeneous, and can manifest as an autosomal dominant or recessive trait. Autosomal recessive congenital cataracts (arCC) have been associated with loci and genes on chromosomes 1p, 1q, 3p, 3q, 6p, 7q, 8p, 9q, 11q, 16q, 17q, 19q, 20p, 21q, and 22q [4–20]. Pathogenic mutations have been reported in EPH receptor A2 (*EPHA2)*, connexin50 (*GJA8)*, FYVE and coiled-coil domain containing 1 (*FYCO1*), glucosaminyl (N-acetyl) transferase 2 (*GCNT2)*, acylglycerol kinase (*AGK*), tudor domain containing 7 (*TDRD7*), crystallin alpha B (*CRYAB*), heat shock transcription factor 4 (*HSF4*), galactokinase 1 (*GALK1*), lens intrinsic membrane protein 2 (*LIM2*), beaded filament structural protein 1 (*BFSP1*), crystallin alpha A (*CRYAA*), lanosterol synthase (*LSS*), crystallin beta B1 (*CRYBB1*), and crystallin beta B3 (*CRYBB3*) [5,6,9,13–15,17–25].

The product of *LIM2* is a 173-amino-acid membrane protein, named MP19, with four transmembrane domains [26]. MP19 is the second most abundant integral membrane protein present in the ocular lens fiber cells of vertebrates [27,28]. It localizes to junction regions of the lens fiber cell membrane as well as throughout the fiber cell membrane, suggesting a role in junction communication [29,30].

To date, only two missense mutations in *LIM2* have been associated with autosomal recessive cataracts. Pras and colleagues reported the c.313T>G (p.F105V) mutation in a family with presenile cortical cataracts and Ponnam and colleagues reported the c.587G>A (p.G154E) mutation in a family with arCC [17,31]. Here, we report a novel missense mutation in *LIM2* in a consanguineous Pakistani family with arCC. This is the first causal mutation for arCC reported in the Pakistani population.

## Materials and Methods

### Patient Recruitment and Clinical Evaluation

A total of 300 plus consanguineous Pakistani families with non-syndromic cataracts were invited to participate in a collaborative study to understand the genetic aspects of arCC. Institutional Review Board (IRB) approval was obtained from the National Eye Institute (Bethesda, MD), Johns Hopkins University School of Medicine (Baltimore, MD), and the National Centre of Excellence in Molecular Biology (Lahore, Pakistan). All participating subjects gave informed written consent consistent with the tenets of the Declaration of Helsinki.

A detailed medical history was obtained by interviewing family members. Ophthalmic examinations, including slit-lamp microscopy, were performed at the Layton Rahmatulla Benevolent Trust (LRBT) Hospital (Lahore, Pakistan). Approximately 10 ml of blood was drawn from all participating members and the samples were stored in 50 ml Sterilin Falcon tubes with 20 mM EDTA. Genomic DNA was extracted as previously described [32,33].

### Genome-Wide Scan

Applied Biosystems MD10 linkage mapping panels (Applied Biosystems, Foster City, CA) were used to complete a genome-wide scan for the family, designated PKCC214. Multiplex polymerase chain reaction (PCR) was completed as previously described [32,33]. PCR products were mixed with a loading cocktail containing 400HD size standards and resolved in a 3100 Genetic Analyzer (Applied Biosystems). Genotypes were assigned using GeneMapper software from Applied Biosystems.

### Linkage Analysis

Two-point linkage analysis was performed using the FASTLINK version of MLINK from the LINKAGE Program Package (provided in the public domain by the Human Genome Mapping Project Resources Centre, Cambridge, UK) [34,35]. Maximum LOD scores were calculated using PLINK (Shaun Purcell, Boston, MA). arCC was analyzed as a fully penetrant trait with an affected allele frequency of 0.001. The marker order and distances between the markers were obtained from the NCBI (National Center for Biotechnology Information, Bethesda, MD) chromosome 19 sequence maps.

### Sanger Sequencing

Primer pairs for individual exons were designed using the Primer3 program. The primer sequences and amplification conditions are provided in Table 1. The PCR amplifications were performed in 10 μl reactions with 20 ng of genomic DNA. The PCR primers for each exon were used for bidirectional sequencing with the BigDye Terminator Ready Reaction mix, according to the manufacturer’s instructions (Thermo Fisher Scientific, Waltham, MA). Sequencing products were dissolved in 10 μl of Formamide (Applied Biosystems) and resolved on an ABI PRISM 3100 Genetic Analyzer (Applied Biosystems). Sequencing results were assembled with ABI PRISM sequencing analysis software, version 3.7, and analyzed with SeqScape software (Applied Biosystems).

**Table 1:** Primer sequences used for the amplification of *LIM2* coding exons.

Exon	Forward	Reverse	Annealing Temperature (°C)
1	GACCATTGTGTAGGGAGGCTTA	GCTTCCTGAGTCCTAGGTGAGA	68
2	CGTCTAGGTCTCCATCTCCTTC	CCCAACTTAACCTTCAAACCAG	68
3	TTTCATCTCAGAGGTAGCAGCA	AGGAGTAAGGGGTGAGAATGGT	68
4	ACCCCTTTCCCCAATCTTAGTT	ACTCCATAGGCCTGGAGTCTTC	68
5	GGATACCCAGGGAGAAAGAGAGT	ACGGGGACTTGAGTCTTCTCAG	68

### Prediction Analysis

The possible impact of an amino acid substitution on the structure of MP19 at the location of the missense mutation was examined with Condel (http://bg.upf.edu/fannsdb), SIFT (http://sift.jcvi.org), PolyPhen-2 (http://genetics.bwh.harvard.edu/pph), Mutation Assessor (http://mutationassessor.org), and FATHMM (http://fathmm.biocompute.org.uk). Evolutionary conservation of the mutated amino acid in MP19 orthologs was examined using the UCSC Genome Browser (http://genome.ucsc.edu).

### Biophysical Characteristics

The hydropathicity, optimized matching hydrophobicity, and polarity of the wild-type and mutant MP19 proteins were examined using ProtScale, a bioinformatics tool on the ExPASy Server (http://www.expasy.org/tools/protscale.html). Similarly, we used ProtScale to compute the isoelectric point (pI) of the wild-type and the mutant MP19 proteins.

### Homology Modeling

Comparative homology modeling of MP19 was performed with predicted 3D structures, retrieved from ModBase, of the wild-type (P55344) and mutant MP19 sequences. The wild-type and mutated protein sequences were also loaded onto Swiss PDB viewer to produce homology models. Hydrogen bonding was incorporated into Swiss PDB Viewer, made available by the Swiss Institute of Bioinformatics (Geneva, Switzerland).

### Real-Time Expression Analysis

The use of mice in this study was approved by the Johns Hopkins Animal Care and Use Committee (ACUC), and all experiments were performed in accordance with a protocol approved by the Johns Hopkins ACUC. Mouse (C57BL/6) lenses were obtained at different developmental stages, including embryonic day 15 (E15), day 18 (E18), at birth (P0), postnatal day 3 (P3), day 6 (P6), day 9 (P9), day 12 (P12), day 14 (P14), day 21 (P21), day 28 (P28), day 42 (P42), and day 56 (P56). Mice lenses were obtained as previously described [32,33]. Mice were exposed to CO_2_ and, after cervical dislocation, the ocular lenses were isolated. Total RNA was isolated with TRIzol Reagent according to the manufacturer’s instructions (Invitrogen, Carlsbad, CA). The quality and quantity of the total RNA were determined on a NanoDrop Lite Spectrophotometer (Thermo Fisher Scientific). First-strand cDNA synthesis was completed using the Superscript III kit according to the manufacturer’s instructions (Invitrogen). Quantitative real-time PCR was performed on a StepOne Real-Time PCR System using commercially available TaqMan expression assays for *Lim2* and *Gapdh*, where the latter was used as an endogenous internal control (Applied Biosystems). The 2^-ΔCT^ method was used to determine the relative expression, normalized to *Gapdh* expression, at each developmental stage.

## Results

**Fig 1:**
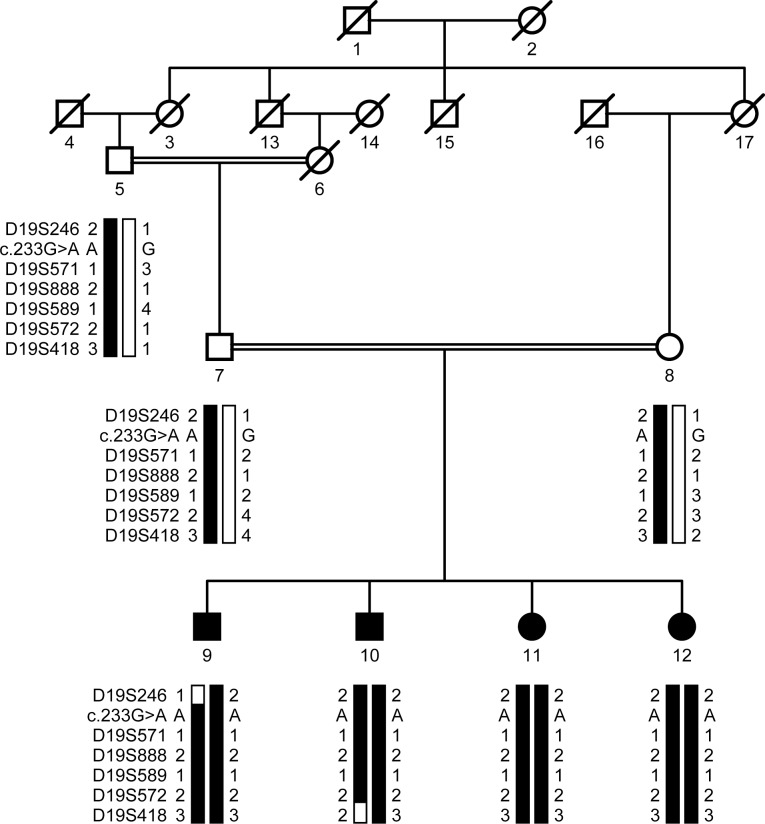
Pedigree drawing of the family PKCC214 with haplotypes of 6 adjacent chromosome 19q microsatellite markers. Alleles that constitute the risk haplotype are shaded black and alleles not co-segregating with cataracts are shown in white. Square: male; circle: female; filled symbol: affected individual; double line between symbols: consanguineous mating; diagonal line through symbol: deceased.

**Fig 2:**
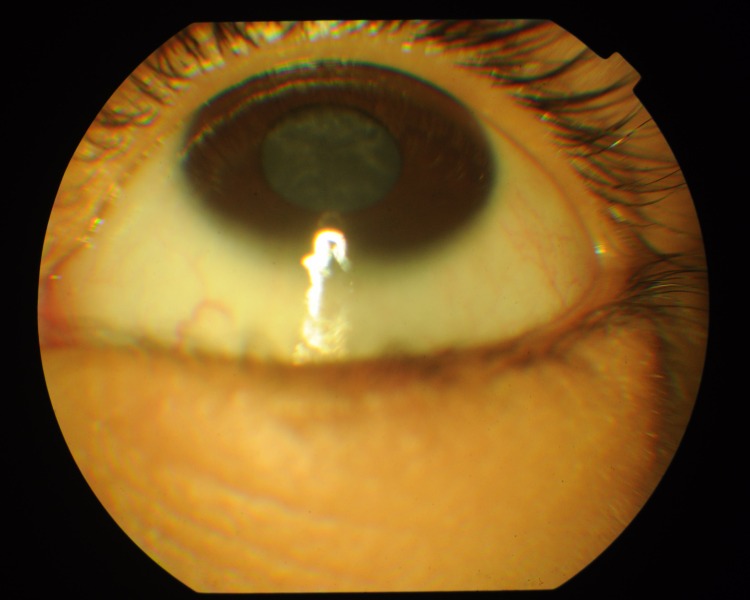
Slit-lamp photograph of affected individual 9 of PKCC214. This photograph depicts a nuclear cataract that developed during infancy.

A consanguineous family, PKCC214, with four affected and three unaffected individuals (Fig 1) was recruited from the Punjab province of Pakistan. A detailed medical history was compiled from interviews with family members and available medical records, which showed that all affected individuals developed cataracts at birth (Table 2). The slit-lamp examinations confirmed the presence of cataracts in the affected individuals exhibiting bilateral nuclear cataracts (Fig 2).

**Table 2:** Clinical characteristics of individuals of PKCC214 who manifest symptoms of autosomal recessive congenital cataracts.

Individual ID	Gender	Age of onset	Age at Enrollment (years)	Visual Acuity (OD/OS)	Clinical Findings
9	M	birth	5	CF/CF	B/L nystagmus
10	M	birth	6	NA	NA
11	F	birth	4	CF/PL	B/L nystagmus; B/L pseudophakia
12	F	birth	8	NA	NA

Age of onset determined by the age at which the first symptoms manifested; CF: counting fingers; PL: perception of light; B/L: bilateral; NA: not available.

We conducted genome-wide scans with the MD10 ABI PRISM panel set and calculated two-point LOD scores according to the method described in previous reports, with some modifications. Linkage was observed with chromosome 19q markers showing maximum LOD scores of 3.25, 3.25, 2.61, and 2.11 at θ = 0 with D19S589, D19S572, D19S571, and D19S888, respectively (Table 3). Haplotype analysis confirmed that the genotypes of affected individuals of PKCC214 were homozygous for D19S571, D19S888, D19S589, and D19S572 while the unaffected individuals were heterozygous. Individual inspection of the haplotypes of PKCC214 supports the results of the linkage analysis and localizes the disease to a 14.5 cM interval between D19S246 and D19S418. A recombination event in affected individual 9 at D19S246 defines the proximal boundary while a recombination event in individual 10 at D19S418 sets the distal boundary (Fig 1). We did not find a significant LOD score with any markers other than those of the region harboring the *LIM2* gene.

**Table 3:** Two-point LOD scores and microsatellite markers used for linkage analysis of PKCC214.

Marker	cM	Mb	0	0.01	0.05	0.09	0.1	0.2	0.3	Z_max_	θ_max_
D19S246	78.08	50.45	**− ∞**	0.07	0.60	0.69	0.69	0.58	0.35	0.69	0.09
D19S571*	84.08	52.79	2.61	2.56	2.36	2.15	2.10	1.57	1.04	2.61	0.0
D19S888*	85.87	53.15	2.11	2.06	1.87	1.67	1.63	1.14	0.64	2.11	0.0
D19S589	87.66	53.30	3.25	3.19	2.96	2.72	2.66	2.04	1.39	3.25	0.0
D19S572*	88.85	53.60	3.25	3.19	2.96	2.72	2.66	2.04	1.39	3.25	0.0
D19S418*	92.56	55.54	**− ∞**	0.66	1.17	1.23	1.22	1.04	0.73	1.23	0.09

An asterisk indicates markers from genome-wide scan.

**Fig 3:**
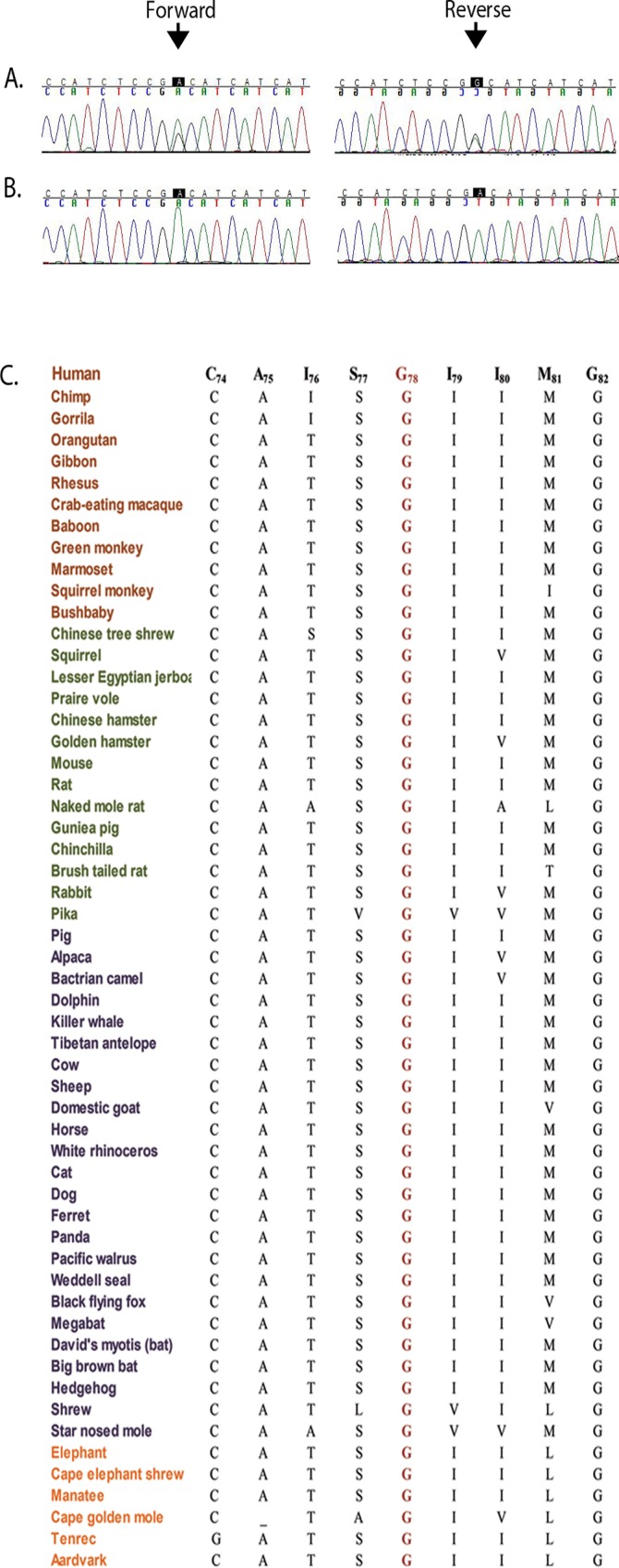
Identification of the pathogenic missense variation responsible for congenital cataracts in PKCC214. **A**) Individual 7 (unaffected; 35 yrs. old) heterozygous carrier and **B**) individual 11 (affected; 4 yrs. old), homozygous for the c.233G>A mutation. The variation results in a non-conservation substitution in MP19: p.G78D. Arrows point to the allele, c.233G, mutated in PKCC214. **C**) Sequence alignment of amino acids illustrating conservation of Gly78 among MP19 orthologs. Brown: Primates; green: Euarchontoglires; purple: Laurasiatheria; and orange: Afrotheria.

The linkage interval harbors more than 100 genes; however, only *LIM2* has previously been associated with cataractogenesis, according to the CatMap database. We sequenced the four coding exons, exon–intron boundaries, and the 5′ and 3′ UTR regions of *LIM2*. A novel missense mutation, c.233G>A, was identified in exon 3 that results in the substitution of aspartic acid for glycine at position 78 (Fig 3A and 3B). This variation segregated with the disease phenotype in PKCC214 and was not present in 192 ethnically matched control chromosomes. Moreover, this variant is not present in the 1000 Genomes database, the ExAC browser, the dbSNP database, or the Exome Variant Server. Importantly, glycine 78 and the amino acids in the immediate neighborhood are well-conserved in MP19 orthologs (Fig 3C). *In silico* analyses with the Condel, SIFT, PolyPhen-2, MutationAssessor, and FATHMM algorithms were all suggestive of the fact that the glycine substitution would not be tolerated and would be damaging to the native structure of the *LIM2*-encoded protein, MP19 (data not shown).

**Fig 4:**
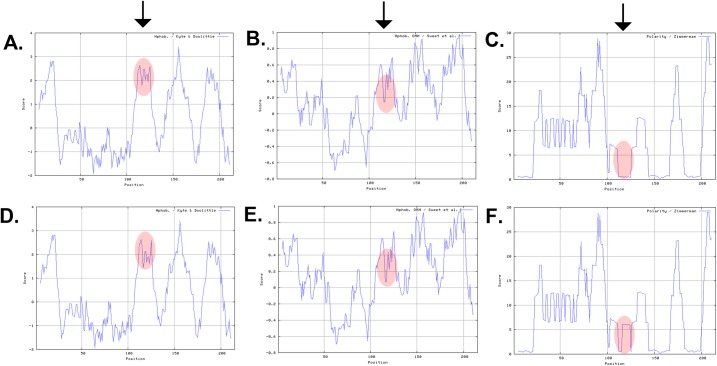
Investigating the physical characteristics of wild-type and mutant MP19. Mutant MP19 (p.G78D) exhibited lower hydropathicity (compare **A** with **D**), lower hydrophobicity (compare **B** with **E**), and higher polarity (compare **C** with **F**). The x-axis represents the position of the amino acids while the y-axis represents the hydropathicity, hydrophobicity, and polarity values in a default window size of 9. Arrows point to the difference in their respective hydropathicity (1^st^ arrow), hydrophobicity (2^nd^ arrow), and polarity (3^rd^ arrow).

Next, we examined the impact of the c.233G>A mutation on the chemical characteristics of MP19. ProtScale software predicted that the mutant MP19 would have lower hydropathicity (Fig 4A and 4D) and lower hydrophobicity (Fig 4B and 4E) compared with the wild type protein. Moreover, the software predicted a higher polarity for mutant MP19 with respect to the wild type protein (Fig 4C and 4F). In parallel, we computed the isoelectric point (pI) of the mutant MP19. We found that the mutation lowers the isoelectric point of the mutant protein (pI: 9.94 vs. pI: 10.04 of the wild type MP19).

**Fig 5:**
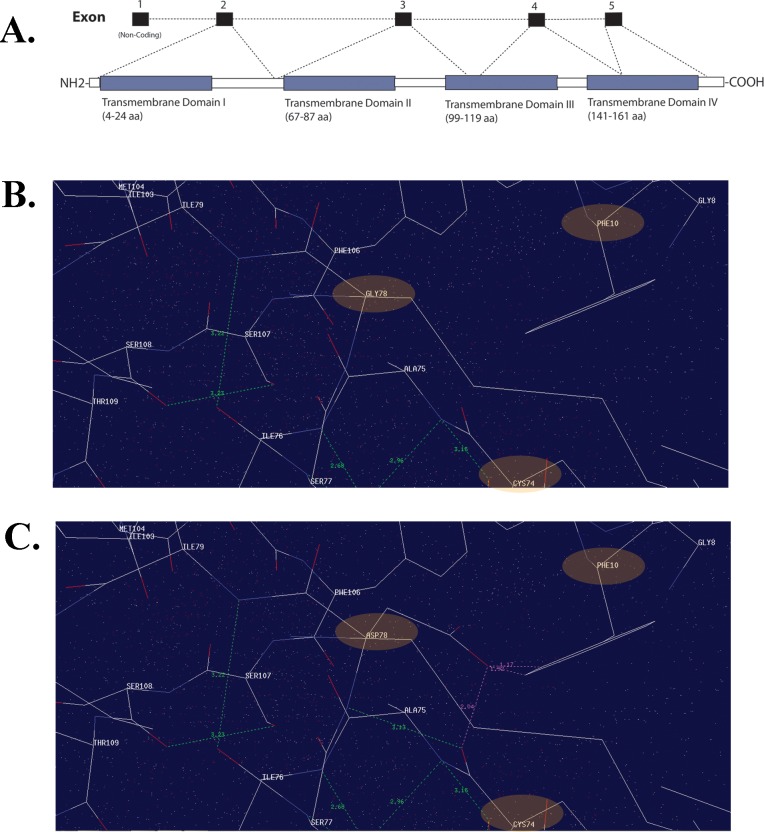
Missense mutation located in the transmembrane domain of MP19 is responsible for congenital cataracts. **A**) *LIM2* graphical illustration showing genomic architecture and MP19 protein structure, including the four transmembrane domains. Note: the topology has been obtained from Maher et al., 2012; Exp Eye Res.103:115–6. Molecular interactions of **B**) wild-type and **C**) mutant MP19 (harboring Gly78Asp) suggesting interactions of the mutant residue (D78) with the phenylalanine (F10) and cysteine (C74) residues.

Computer modeling predicts MP19 to have four helical transmembrane domains, and two extracellular and three cytoplasmic topological domains (Fig 5A) [36]. To investigate the effect of the p.G78D mutation on the normal folding and protein conformation, we compared the ModBase-predicted 3D structures of the wild-type (P55344) and mutant MP19 (Fig 5B and 5C). The comparison suggests that the aspartic acid side chain of mutant MP19 forms bonds with the amino acids phenylalanine 10 (F10) and cysteine 74 (C74) (Fig 5C). The bond formation between the mutated amino acid, D78, and F10 of the first transmembrane domain is likely to disrupt the membrane-associated topology of the native protein structure.

**Fig 6:**
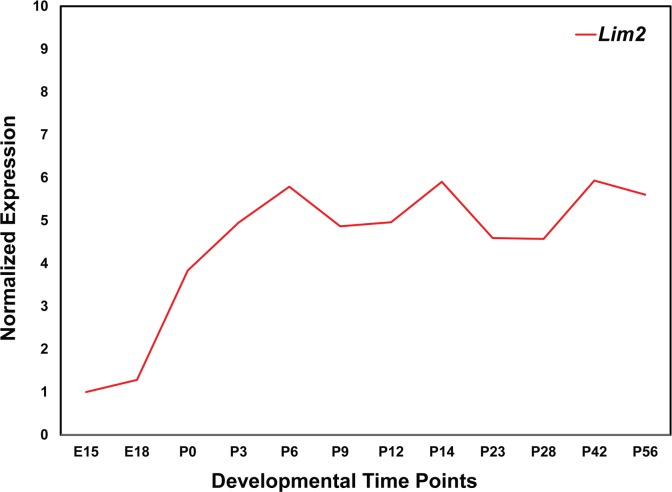
Expression profile of lens intrinsic membrane protein 2 (*Lim2*) in the developing mouse lens. The expression of *Lim2* at different developmental time points was normalized to *Gapdh*. The x-axis and y-axis represent developmental time points and normalized expression of *Lim2* mRNA, respectively.

As a first step in understanding the physiological significance of *LIM2* in the development of the ocular lens, we explored the expression of *Lim2* in our recently published mouse lens transcriptome. *Lim2* is expressed as early as embryonic day 15 [37]. Low levels of expression were observed in the embryonic stages; however, relatively higher expression was observed at the postnatal time points [37]. To further investigate the expression profile of *Lim2* in the mouse lens, we performed quantitative real-time PCR analysis at six additional postnatal time points and used the expression level at E15 as a reference for the comparison of the subsequent 11 time points. As shown in Fig 6, consistent with our previously published results, *Lim2* is expressed at lower levels at embryonic timepoints and expression increases considerably during early postnatal stages, remaining steady afterward. These data suggest a critical role for *LIM2* in the development of the ocular lens and the maintenance of its transparency.

## Discussion

Here, we report a novel missense mutation in *LIM2* that segregates with autosomal recessive congenital cataracts in a consanguineous Pakistani family. Slit-lamp examination of the affected family members revealed nuclear cataracts. The medical records available to us indicate the cataracts developed during infancy. Linkage analysis determined the critical interval of 14.5 cM on chromosome 19, with a maximum LOD score of 3.25 at the recombination fraction of 0. Sequencing the *LIM2* gene identified a missense mutation, c.233G>A. This single-nucleotide variation resulted in a change from the nonpolar, smallest amino acid, glycine, to the larger, negatively charged aspartic acid. The glycine at position 78 is strongly conserved across different species. While the maximum LOD score of 3.25 is slightly higher than the traditional limiting value of 3.0, it represents the maximum value obtainable with this family. In addition, the lack of any LOD scores above 1.5 in the remainder of the genome-wide scan supports the localization of the cataract locus to chromosome 19q13.

Steel and colleagues reported the association of *LIM2* with lens opacity in To3 transgenic mice, identifying a single-base change in the first coding exon of *Lim2* that results in a glycine-to-valine substituion at amino acid 15: c.44G>T (p.G15V) [38]. Chen and colleagues demonstrated that, in contrast to wild-type MP19 proteins, which localize to the cell membrane, mutant MP19^TO3^ proteins are retained within subcellular compartments [39]. To understand the physiological significance of *LIM2* in lens morphogenesis, Shi and colleagues used both scanning electron and confocal microscopy to assess the contribution of *Lim2* to mouse lens tissue architecture, and concluded that Mp19 deficiency distinctly altered the characteristic morphology of mouse lens fiber cells. The researchers argued that their data suggested a critical role for Mp19 in the maintenance of cytoskeletal integrity, cell morphology, and intercellular communication in the mouse lens [40].

Previously, only two causal mutations in *LIM2* have been identified in patients with cataracts; the mutation identified here is now a third avenue of insight into the role of *LIM2* in cataractogenesis. Interestingly, the patients in each study exhibit different clinical characteristics than those of the other two studies, despite the cataracts being caused by mutations in the same gene. The patients reported by Pras and colleagues developed cataracts in their presenile years, which is in sharp contrast to members of PKCC214 and the patients reported by Ponnam and colleagues, who all exhibited cataractogenesis with early onset (Table 4). Moreover, the patients of Pras et al. exhibited no additional ocular anomalies, while cataract development in PKCC214 and the patients reported by Ponnam et al. was concurrent with nystagmus (Table 4). Last but not least, the cataracts themselves are morphologically different in all three reports. The patients in the family reported by Pras et al. exhibited cortical bluish and white opacifications in concentric layers, with nuclear opacities emerging from prominent sutures (Table 4). In contrast, patients reported by Ponnam et al. had total cataracts while members of PKCC214 manifested nuclear cataracts (Tables 2 & 4).

**Table 4:** Summary of mutations identified in *LIM2* associated with cataractogenesis.

No	Nucleotide Change	Amino Acid Change	Exon	Cataractogenesis Onset	Cataract Phenotype	Additional Phenotypes	Ethnicity	Reference
1	c.313T>G	p.F105V	3	Presenile	pulverulent cortical/ nuclear	-	Iraqi/Arab	[17]
2	c.587G>A	p.G154E	4/5	Congenital	total	nystagmus; dense amblyopia	Indian	[31]
3	c.233G>A	p.G78D	3	Congenital	nuclear	nystagmus	Pakistani	current study

The mechanism of cataractogenesis remains elusive, including the particular effects of missense mutations in *LIM2*. It is notable that all three mutations affect conserved amino acids, and that each missense change has an autosomal recessive loss of function mode of inheritance. Interestingly, all three mutated amino acids reside in transmembrane domains of MP19, according to the topology reported by Maher and colleagues [41]. The missense changes reported by Pras et al. and Ponnam et al. [c.313T>G (p.F105V) and c.587G>A (p.G154E)] reside in the third and fourth transmembrane domains of MP19, while the missense change in PKCC214 [c.233G>A (p.G78D)] is predicted to reside in the second transmembrane domain. Given that all of the missense changes are responsible for cataractogenesis, it is tempting to speculate that the altered topology of MP19 (a likely result of missense mutations compromising conserved regions) affects translocation to the cell membrane or the protein’s physiological function at the membrane. Nonetheless, detailed mechanistic analysis of these mutant proteins, especially their relationships to one another, will contribute significantly in discovering the pathomechanism of cataractogenesis.

In conclusion, we report a missense mutation in the sequence of *LIM2* encoding the second transmembrane domain of MP19 that causes autosomal recessive congenital cataracts. Identification of the genetic origins of cataractogenesis will help us to better understand the biology of the ocular lens, including mechanistic details of the maintenance of lens transparency.

## Supporting Information

S1 File The ARRIVE checklist has been completed for the manuscript. (PDF)
